# Prehospital transdermal glyceryl trinitrate in patients with ultra-acute presumed stroke (RIGHT-2): effects on outcomes at day 365 in a randomised, sham-controlled, blinded, phase III, superiority ambulance-based trial

**DOI:** 10.1136/bmjno-2023-000424

**Published:** 2023-06-27

**Authors:** Lisa J Woodhouse, Jason P Appleton, Sandeep Ankolekar, Timothy J England, Grant Mair, Keith Muir, Christopher I Price, Stuart Pocock, Marc Randall, Thompson G Robinson, Christine Roffe, Else C Sandset, Jeffrey L Saver, Aloysius Niroshan Siriwardena, Nikola Sprigg, Joanna M Wardlaw, Philip M Bath, the RIGHT-2 Investigators

**Affiliations:** 1 Stroke Trials Unit, Mental Health and Clinical Neurosciences, School of Medicine, University of Nottingham, Nottingham, UK; 2 Stroke, Nottingham University Hospitals NHS Trust, Nottingham, UK; 3 Department of Neurology, King's College Hospital NHS Trust, London, UK; 4 Vascular Medicine, Division of Medical Sciences and GEM, Royal Derby Hospital, Derby, UK; 5 UK Dementia Research Institute, The University of Edinburgh Centre for Clinical Brain Sciences, Edinburgh, UK; 6 Neurology, University of Glasgow, Glasgow, UK; 7 Institute of Neuroscience, Newcastle University, Newcastle upon Tyne, UK; 8 Department of Medical Statistics, London School of Hygiene & Tropical Medicine, London, UK; 9 Department of Neurology, Leeds Teaching Hospitals NHS Trust, Leeds, UK; 10 Department of Cardiovascular Sciences, and NIHR Biomedical Research Unit for Cardiovascular Diseases, University of Leicester, Leicester, UK; 11 Institute for Science and Technology in Medicine, Keele University, Keele, UK; 12 Department of Neurology, Oslo University Hospital, Oslo, Norway; 13 Research and Development, Norwegian Air Ambulance Foundation, Oslo, Norway; 14 Department of Neurology and Comprehensive Stroke Center, David Geffen School of Medicine, Los Angeles, California, USA; 15 Community and Health Research Unit, University of Lincoln, Lincoln, UK

**Keywords:** STROKE, CEREBROVASCULAR DISEASE, RANDOMISED TRIALS, CEREBRAL BLOOD FLOW

## Abstract

**Background:**

The Rapid Intervention with Glyceryl Trinitrate in Hypertensive Stroke Trial-2 (RIGHT-2) reported no overall treatment difference between glyceryl trinitrate (GTN) and sham at day 90. Here we assess participants’ outcomes 1 year after randomisation.

**Methods:**

RIGHT-2 was an ambulance-based prospective randomised controlled trial where patients with presumed stroke and systolic blood pressure (BP) of >120 mm Hg received either GTN (5 mg/day) or sham patch. Centralised blinded telephone follow-up was performed at days 90 (primary endpoint) and 365 (secondary endpoint). The lead outcome was dependency assessed with the modified Rankin Scale (mRS).

**Results:**

1149 patients were recruited to RIGHT-2 between October 2015 and May 2018, and 1097 (95.5%) had outcome data recorded at day 365. At baseline, the patients were; female (48%), had a mean age of 73 (15) years, BP of 162 (25)/92 (18) mm Hg, onset to randomisation of 70 (45–115) min, diagnosis of ischaemic stroke (52%), intracerebral haemorrhage (ICH) (13%), transient ischaemic attack (TIA) (9%) and mimics (26%). There was no effect of GTN on mRS score at day 365 in participants with confirmed stroke/TIA (adjusted common odds ratio (acOR) 1.10, 95% CI 0.86 to 1.42) or in all patients. In patients randomised to GTN, mRS at day 365 tended to be worse in those with ICH (acOR 1.65, 95% CI 0.84 to 3.25) and better in those with a mimic diagnosis (acOR 0.53, 95% CI 0.33 to 0.84).

**Conclusion:**

At 1 year post randomisation, dependency did not differ between GTN and sham treatment in either the target population or overall. In prespecified subgroup analyses, GTN was associated with reduced dependency in participants with a final diagnosis of mimic and a non-significant worse outcome in participants with ICH.

**Trial registration number:**

ISRCTN26986053.

**WHAT IS ALREADY KNOWN ON THIS TOPIC**

- The Rapid Intervention with Glyceryl trinitrate in Hypertensive stroke Trial-2 (RIGHT-2) reported no evidence of a treatment difference between glyceryl trinitrate (GTN) and sham on outcomes collected at day 90.

**WHAT THIS STUDY ADDS**

- As poststroke recovery evolves, although more slowly, between 3 months and 12 months, the assessment of RIGHT-2 outcomes at 1 year provides an important, fuller delineation of the effect of GTN.

**HOW THIS STUDY MIGHT AFFECT RESEARCH, PRACTICE OR POLICY**

- This study reports the long-term outcomes of the RIGHT-2 trial. Follow-up beyond 3 months is not typical in acute stroke trials but is increasingly recommended, especially in patients with severe and/or haemorrhagic stroke, where longer periods of follow-up to 6, 12 or even 18 months may be needed to see significant improvements in functional outcome and differences between treatment groups.

## Introduction

High blood pressure (BP) is common in acute stroke and a predictor of poor outcome, and so a target for therapeutic lowering.^1^ Vascular nitric oxide (NO) levels are low in acute stroke and are associated with a poor outcome, so replacing NO, a cerebral and systemic vasodilator, with a donor might be beneficial.^2 3^ Preclinical stroke studies found that NO donors improved regional cerebral blood flow and reduced stroke lesion size if administered rapidly^4 5^; further, glyceryl trinitrate (GTN) improved functional outcome in the phase II Rapid Intervention with Glyceryl Trinitrate in Hypertensive Stroke Trial-2 (RIGHT, with randomisation by paramedics within 4 hours of stroke)^6^ and a prespecified subgroup analysis of the phase III hospital-based Efficacy of Nitric Oxide in Stroke trial (with randomisation within 6 hours of stroke).^7 8^ Individual patient data meta-analyses of trials of GTN suggested that very early administration was beneficial in both ischaemic stroke (IS) and intracerebral haemorrhage (ICH), and reduced death, disability, cognitive impairment, mood disturbance and poor quality of life.^9 10^

However, the subsequent phase III RIGHT-2 trial assessed the safety and efficacy of GTN when administered before hospital admission and within 4 hours of stroke onset and found no overall benefit on the primary outcome time point of 3 months post stroke.^11^ As poststroke recovery does evolve, although more slowly, between 3 months and 12 months, assessment of RIGHT-2 outcomes at 1 year provides an important evaluation of whether early, short-term administration of GTN has an impact on long-term outcome. Although follow-up beyond 90 days is not typical in trials of acute stroke, it is becoming increasingly recommended, especially in severe stroke. The concept behind this is that a longer period of follow-up may be required to see if significant differences between treatment groups manifest post 90 days. It is also important to note though that longer follow-up could potentially add noise from extraneous events.

## Methods

RIGHT-2 was a prospective, multicentre, paramedic-delivered, ambulance-based, randomised, sham-controlled, participant and outcome blinded phase III trial in adult patients with ultra-acute presumed stroke in the UK.^12^ Further information regarding the sample size calculation, randomisation and blinding has already been published elsewhere.^12^ Patients were eligible for inclusion following an emergency 999 call for presumed stroke if they presented within 4 hours of their symptoms to a trial-trained paramedic from a participating ambulance service, and it was possible for them to be taken to a participating hospital. The patient had to have a Face–Arm–Speech–Time Test (FAST) score of 2 or 3 and a systolic BP of ≥120 mm Hg. However, if the patient was from a nursing home, had reduced consciousness (Glasgow Coma Scale score <8), hypoglycaemia (capillary glucose <2.5 mmol/L) or a witnessed seizure, then the patient was excluded. Included patients were randomly assigned 1:1 to receive transdermal GTN (5 mg as Transiderm-Nitro 5) or sham (DuoDERM hydrocolloid dressing). The first treatment was administered immediately after randomisation in the ambulance, and further treatments were given for up to 3 days in the hospital. The final diagnosis of IS, ICH, mimic or transient ischaemic attack (TIA) was made at the hospital following clinical review and brain imaging (mostly with CT); all scans underwent central adjudication as described previously.^11^

### Outcomes

Centralised blinded telephone follow-up was performed by a trained assessor masked to treatment allocation for patients at 365 days post randomisation. If the participant was aphasic or for some other reason incapable of completing the follow-up, then information was collected from a relative or carer. When the participant/relative/carer could not be contacted by telephone, a questionnaire covering the same outcome measures was sent by post. Outcome assessments covered dependency (seven-level modified Rankin Scale (mRS)), activities of daily living (Barthel Index, BI), cognition (modified telephone Mini-mental State Examination and Telephone Interview for Cognitive Status–Modified (TICS-m)), categorical verbal fluency using animal naming, health-related quality of life (European Quality of Life Five-Dimensional Three-Level Health Status Utility Value (EQ-5D-HSUV) and European Quality of Life Visual Analogue Scale) and mood (abbreviated Zung Depression Score (ZDS)). Other outcome measures included home time, calculated as the number of days between discharge and day 365, and all-cause mortality.

### Statistical analysis

Analyses were hierarchical, first in participants with a confirmed stroke (IS and ICH) or TIA (cohort 1), and then in all who were randomised (intention-to-treat, cohort 2) according to the published statistical analysis plan.^13^ The mRS score was analysed using ordinal logistic regression, adjusted for age, sex, premorbid mRS, baseline FAST score, systolic BP and time from symptom onset to randomisation. Unadjusted, per-protocol and imputed (with missing mRS scores estimated using multiple regression-based imputation) sensitivity analyses were also performed for completeness. Heterogeneity of the treatment effect on the mRS score was assessed in prespecified subgroups by adding an interaction term to an adjusted ordinal logistic regression model. All-cause mortality was assessed using adjusted Cox proportional hazard models. Other outcomes were analysed using adjusted binary logistic regression, ordinal logistic regression and multiple linear regression, with adjustment as previously mentioned. A prespecified global outcome (including data from the mRS, BI, ZDS, TICS-m and EQ-5D-HSUV) was analysed using the Wei-Lachin test.^14^ Participants who did not receive their assigned treatment, did not adhere to the protocol or were eventually diagnosed as mimics were still followed up in full at day 365 and were included in the analyses.

## Results

Of the 1149 randomised participants,^15^ 1097 (95.5%; GTN 541 and sham 556) had outcome data recorded at day 365, with a final diagnosis of stroke (IS and ICH) or TIA present in 818 (GTN 415 and sham 403) (table 1). Demographic and clinical characteristics were similar in the two treatment groups across the whole trial population and in patients with stroke or TIA; overall, the mean age was 72.6 (14.5) years; women comprised 48%; maximum FAST score=3 (60%); GCS score <14 (26%); and the final diagnosis of the qualifying event: IS (52%), ICH (13%), TIA (9%) and a stroke/TIA-mimicking condition (26%). Common causes of neurovascular mimics included seizure (17%), migraine (16%) and functional symptoms (15%).

**Table 1:** Baseline patient characteristics in the ambulance and at hospital admission in patients with confirmed stroke or TIA (target population, cohort 1) and all participants (intention-to-treat population, cohort 2) with outcome data at day 365

	Cohort 1			Cohort 2		
	All	GTN	Sham	All	GTN	Sham
Ambulance data/prerandomisation						
Number of patients	818	415	403	1097	541	556
Age (years)	74.6 (12.5)	73.8 (12.8)	75.5 (12.1)	72.6 (14.5)	72.4 (14.5)	72.9 (14.5)
Sex (male) (%)	440 (54)	226 (54)	214 (53)	568 (52)	280 (52)	288 (52)
Time from onset to randomisation (min)	70 (45–105)	70 (45–105)	70 (45–107)	70 (45–115)	70 (45–111)	72 (45–115)
ECG, Atrial Fibrillation/flutter (%)	155 (23)	79 (24)	76 (22)	184 (21)	90 (21)	94 (21)
Systolic BP (mm Hg)	163.1 (24.6)	162.9 (24.3)	163.2 (25)	162.1 (25)	161.2 (24.5)	162.9 (25.6)
Diastolic BP (mm Hg)	91.6 (18.4)	91.9 (18.9)	91.3 (17.8)	91.3 (17.8)	91.3 (18.4)	91.4 (17.2)
Heart rate (beats/min)	81.7 (18.5)	81.4 (18.5)	82 (18.6)	82.1 (18.6)	81.5 (17.9)	82.7 (19.3)
Glasgow Coma Scale score <14 (%)	221 (27)	119 (29)	102 (25)	288 (26)	155 (29)	133 (24)
FAST score=3 (%)	526 (64)	264 (64)	262 (65)	663 (60)	328 (61)	335 (60)
Hospital admission/post treatment						
Number of patients with data	818	415	403	1097	541	556
Ethnic group, non-white (%)	75 (9)	34 (8)	41 (10)	107 (10)	48 (9)	59 (11)
Premorbid mRS score >2 (%)	138 (17)	73 (18)	65 (16)	211 (19)	110 (21)	101 (18)
Medical history (%)						
Hypertension	481 (59)	244 (59)	237 (59)	616 (57)	303 (57)	313 (57)
Diabetes mellitus	161 (20)	78 (19)	83 (21)	218 (20)	105 (20)	113 (21)
Previous stroke	177 (22)	95 (23)	82 (20)	258 (24)	131 (25)	127 (23)
Ischaemic heart disease	134 (16)	63 (15)	71 (18)	189 (18)	92 (17)	97 (18)
Smoking, current	106 (16)	57 (17)	49 (15)	157 (18)	82 (19)	75 (17)
Qualifying event (%)						
Ischaemic stroke	573 (70)	288 (69)	285 (71)	573 (52)	288 (53)	285 (51)
Intracerebral haemorrhage	141 (17)	71 (17)	70 (17)	141 (13)	71 (13)	70 (13)
TIA	103 (13)	55 (13)	48 (12)	103 (9)	55 (10)	48 (9)
Mimics	0 (0)	0 (0)	0 (0)	279 (25)	126 (23)	153 (28)
OCSP TACS (%)	301 (38)	155 (39)	146 (38)	339 (34)	168 (34)	171 (34)
NIHSS score (/42)	9 (4–16)	9 (4–16)	9 (4–16)	8 (4–15)	8 (3–16)	7 (4–15)
Reperfusion therapy (%)						
Intravenous thrombolysis	273 (48)	144 (50)	129 (45)	273 (48)	144 (50)	129 (45)
Thrombectomy	24 (4)	7 (2)	17 (6)	24 (4)	7 (2)	17 (6)

Data are number (%), mean (SD) and median (IQR).

FAST, Face–Arm–Speech–Time Test; GTN, glyceryl trinitrate; mRS, modified Rankin Scale; NIHSS, National Institutes of Health Stroke Scale; OCSP, Oxfordshire Community Stroke Project; TACS, total anterior circulation stroke; TIA, transient ischaemic attack.

### Clinical outcomes

**Figure 1:**
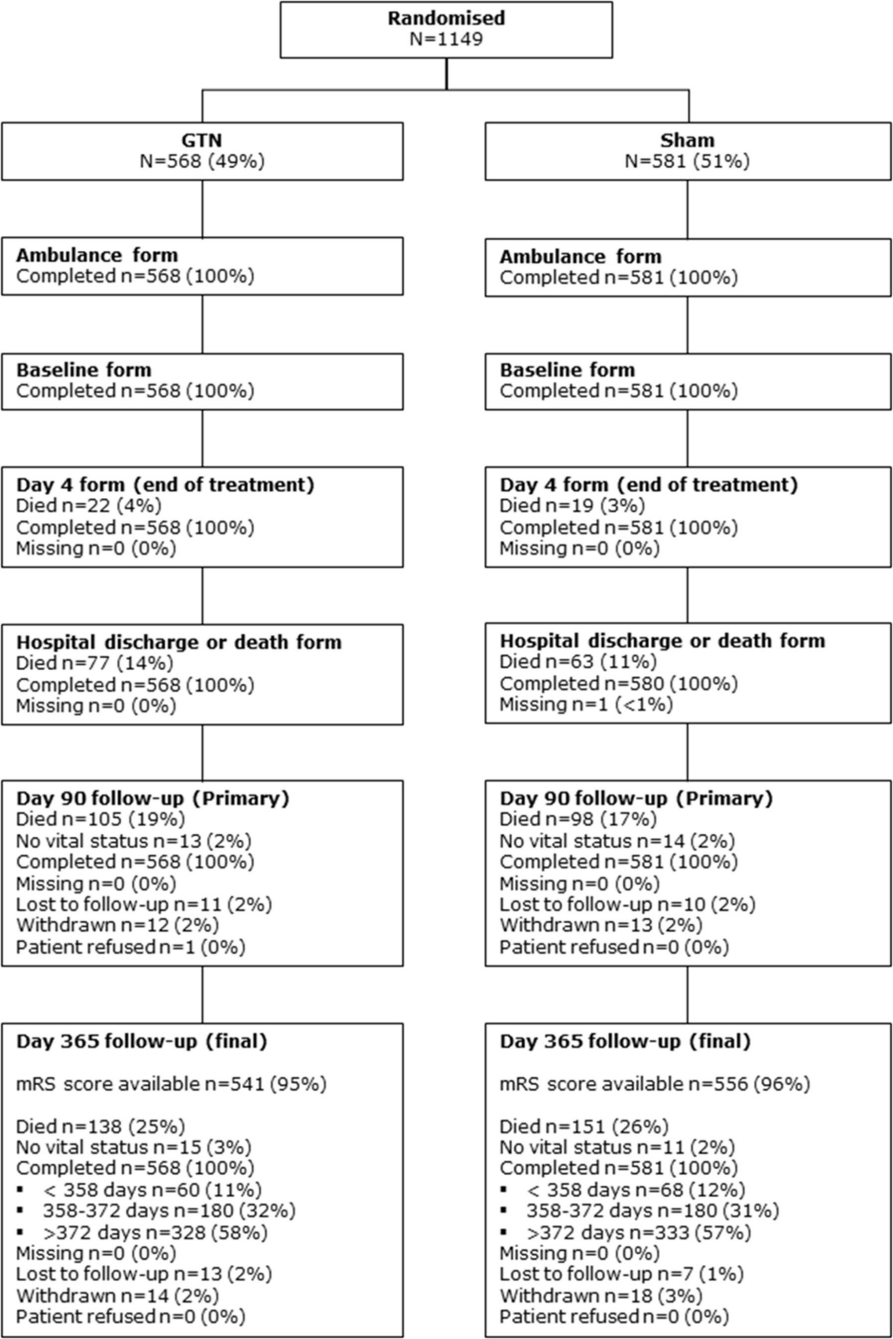
Consolidated Standards of Reporting Trials diagram. GTN, glyceryl trinitrate; mRS, modified Rankin Scale.

Vital status and mRS were available in 1123 (98%) and 1097 (95%) participants, respectively; there was no differential loss to follow-up or withdrawals between the treatment groups (figure 1). Blinding was maintained with 96% of participants unable to identify which medication they had received.

**Figure 2:**
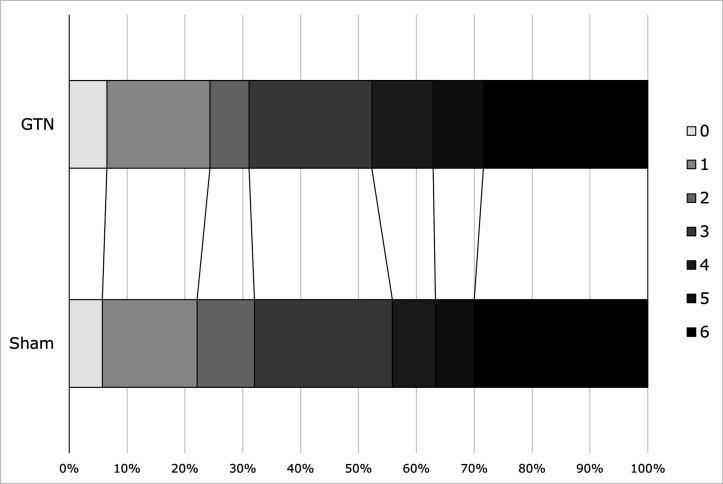
Distribution of mRS score at day 365 for GTN versus sham in patients with confirmed stroke or TIA (target population, cohort 1). GTN, glyceryl trinitrate; mRS, modified Rankin Scale; TIA, transient ischaemic attack.

**Figure 3:**
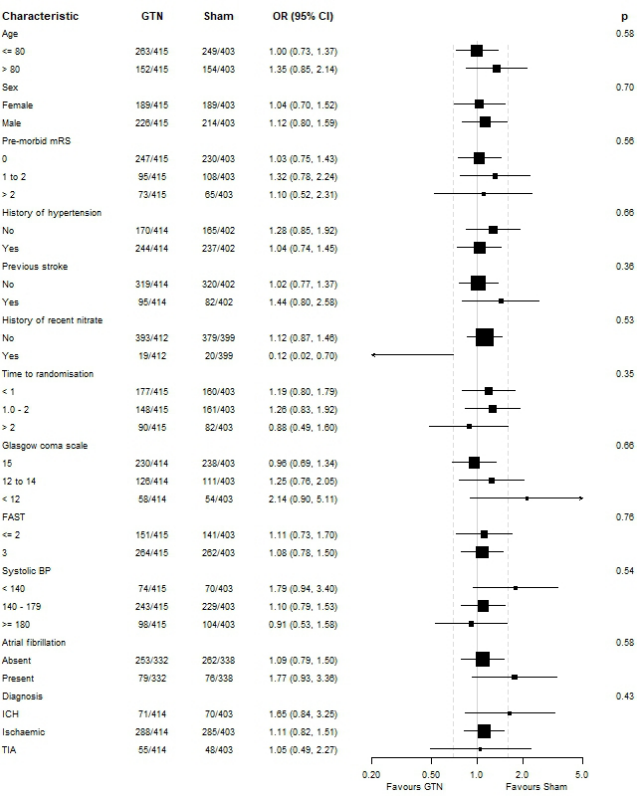
Forest plot for patients with confirmed stroke or TIA (target population, cohort 1). BP, blood pressure; FAST, Face–Arm–Speech–Time Test; GTN, glyceryl trinitrate; ICH, intracerebral haemorrhage; TIA, transient ischaemic attack.

In participants with confirmed stroke and TIA (cohort 1/target population), there was no evidence of an effect of GTN on dependency at 365 days in comparison with sham (GTN 3 (IQR 2–6) versus sham 3 (IQR 2–6), acOR 1.10, 95% CI 0.86 to 1.42, p=0.44; table 2 and figure 2). In sensitivity analyses, there was no difference in mRS score when compared as mean difference, proportions with dependency or death (mRS score >2), ordinal mRS in the *per protocol* population or when data were imputed for participants without a recorded mRS at day 365 (table 2). When assessed in pre-specified subgroups, no significant effect was detected (figure 3). When assessed in components of the target population (cohort 1), mRS score did not differ between GTN and sham in participants with all stroke (IS and ICH), IS alone or TIA. However, GTN was associated with a non-significantly worse outcome in patients with a final diagnosis of ICH (GTN median 6 (IQR 4–6) vs sham 5 (3–6), acOR 1.65, 95% CI 0.84 to 3.25); p=0.15; n=141).

**Table 2:** Primary and secondary outcomes at day 365 in patients with confirmed stroke or TIA (target population, cohort 1) and all participants (intention-to-treat population, cohort 2)

Outcome	Cohort 1				Cohort 2			
	N	GTN	Sham	OR/MD (95% CI), P adjusted	N	GTN	Sham	OR/MD (95% CI), P adjusted
mRS score (/6)								
All (mITT)	818	3 (2–6)	3 (2–6)	1.10 (0.86–1.42), p=0.44	1097	3 (1–6)	3 (2–6)	0.94 (0.76–1.18), p=0.61
Sensitivity analyses								
All (PP)	706	3 (2–6)	3 (2–6)	1.12 (0.85–1.47), p=0.42	956	3 (1–6)	3 (1,6)	0.99 (0.78– 1.26), p=0.95
All (MI)	852	3 (2–6)	3 (2,6)	1.10 (0.86–1.41), p=0.46	1149	3 (1–5)	3 (2,6)	0.97 (0.78–1.2), p=0.76
Mean mRS	818	3.5 (2.0)	3.5 (2.0)	0.08 (−0.14 to 0.30), p=0.49	1097	3.3 (2–1)	3.3 (2.1)	−0.07 (−0.27 to 0.12), p=0.48
mRS score >2 (%)	818	286 (69)	274 (68)	1.22 (0.86–1.72), p=0.27	1097	354 (65)	369 (66)	1.00 (0.74–1.34), p=0.98
Unadjusted	818	3 (2–6)	3 (2–6)	1.00 (0.79–1.28), p=0.98	1097	3 (1–6)	3 (2–6)	0.96 (0.78–1.18), p=0.67
Subgroups								
Stroke (mITT)	714	4 (2–6)	3 (2–6)	1.17 (0.89–1.54), p=0.25	714	4 (2–6)	3 (2–6)	1.17 (0.89–1.54), p=0.25
ICH (ITT)	141	6 (4–6)	5 (3–6)	1.65 (0.84–3.25), p=0.15	–	–	–	–
IS and TIA (ITT)	676	3 (1–5)	3 (2–6)	1.04 (0.79–1.38), p=0.77	–	–	–	–
TIA	103	1 (1–3)	2 (1–3)	1.05 (0.49–2.27), p=0.90	–	–	–	–
Mimics (ITT)	–	–	–	–	**279**	**3 (0–4)**	**3 (1–5)**	**0.53 (0.33–0.84), p=0.007**
Secondary outcomes								
Death (%)								
Stroke and TIA	835	118 (28)	121 (29)	0.99 (0.77–1.29), p=0.96	–	–	–	–
All	–	–	–	–	1123	138 (25)	151 (26)	0.94 (0.74–1.19), p=0.59
Stroke	728	113 (31)	116 (32)	1.01 (0.78–1.32), p=0.93	–	–	–	–
TIA	106	4 (7)	5 (10)	3.18 (0.10–98.74), p=0.51	–	–	–	–
Mimics	–	–	–	–	288	20 (16)	30 (19)	0.63 (0.34–1.16), p=0.14
Disposition (%)	791	1 (1–3)	1 (1–3)	1.1 (0.79–1.52), p=0.57	1043	1 (1–3)	1 (1,3)	0.99 (0.74–1.32), p=0.94
EQ-5D-HSUV (/1)	774	0.3 (0.4)	0.4 (0.4)	−0.02 (−0.07 to 0.03), p=0.38	1020	0.4 (0.4)	0.4 (0.4)	−0.01 (−0.05 to 0.04), p=0.75
Barthel Index (/100)	777	52 (46.4)	52.8 (46.7)	−2.21 (−7.50 to 3.08), p=0.41	1023	55.4 (45.9)	55.7 (46.1)	0.13 (−4.45 to 4.71), p=0.96
TICS-m	447	10.3 (13)	10.3 (12.6)	0.30 (−1.47 to 2.08), p=0.74	572	11.2 (13)	10.7 (12.5)	0.66 (−0.92 to 2.23), p=0.41
ZDS (/100)	488	76 (28.9)	75 (28.7)	0.81 (−3.24 to 4.86), p=0.69	636	74.4 (28.9)	74.2 (28.6)	0.55 (−3.05 to 4.16), p=0.76
Home time (days)	660	229.4 (196)	236.9 (190.3)	−11.0 (−35.0 to 12.91), p=0.37	874	260.3 (197.4)	257.9 (185.6)	6.95 (−14.0 to 27.86), p=0.52
Global analysis	447	–	–	0.00 (−0.08 to 0.08), p=0.96	572	–	–	−0.01 (−0.08 to 0.05), p=0.71

Data are number (%), mean (SD), and median (IQR), unless otherwise stated.

Bold values denote that the result is statistically significant.

EQ-5D-HSUV, European Quality of Life Five-Dimensional Three-Level Health Status Utility Value; ICH, intracerebral haemorrhage; IS, ischaemic stroke; ITT, intention to treat; MI, multiple regression-based imputation; mITT, modified intention to treat; mRS, modified Rankin Scale; PP, per protocol; TIA, transient ischaemic attack; TICS-m, Telephone Interview for Cognitive Status–Modified; ZDS, Zung Depression Score.

**Figure 4:**
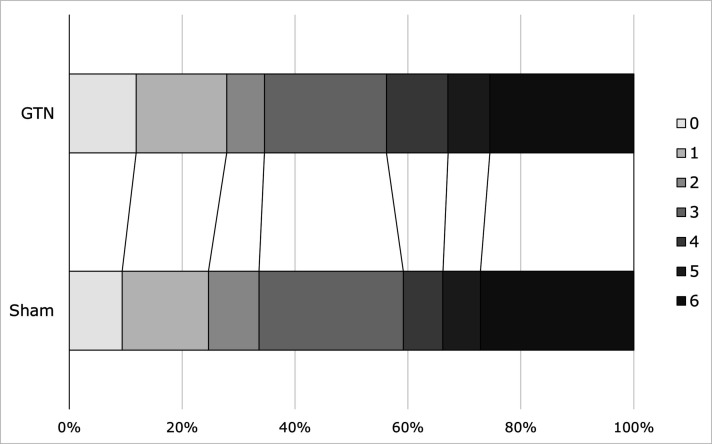
Distribution of mRS score at day 365 for GTN versus sham in all participants (intention-to-treat population, cohort 2). GTN, glyceryl trinitrate;

**Figure 5:**
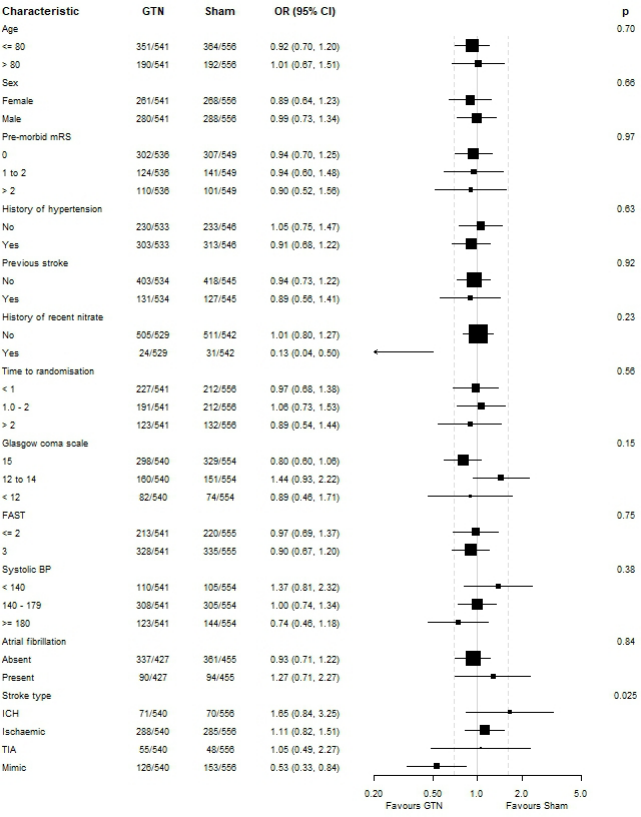
Forest plot in all participants (intention-to-treat population, cohort 2). BP, blood pressure; FAST, Face–Arm–Speech–Time Test; GTN, glyceryl trinitrate; ICH, intracerebral haemorrhage; TIA, transient ischaemic attack.

The analysis of all patients in the trial (cohort 2/ITT) also showed that mRS did not differ between GTN and sham in the primary analysis or in any sensitivity analysis (table 2 and figure 4). In predefined subgroups, there was a significant interaction by final diagnosis (figure 5); in contrast to the effect of GTN in stroke or TIA (see above), GTN was associated with less dependency than sham in patients with a mimic diagnosis (non-stroke/TIA) (acOR 0.53, 95% CI 0.33 to 0.84, p=0.007).

**Figure 6:**
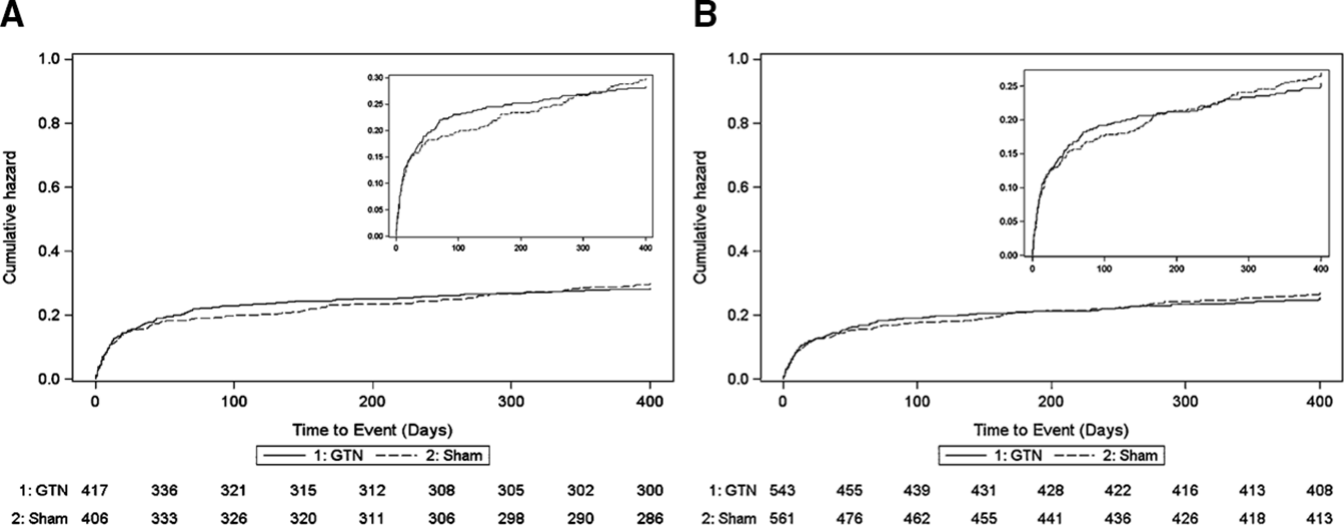
Deaths as cumulative hazard in (A) patients with confirmed stroke or TIA (target population, cohort 1), or (B) all participants (intention-to-treat population, cohort 2). GTN, glyceryl trinitrate; TIA, transient ischaemic attack

There was no difference between GTN and sham in secondary outcomes at day 365 in either cohort 1 or 2 (table 2). Similarly, rates of death by day 365 did not differ between the treatment groups in either cohort (figure 6).

## Discussion

In this prospective follow-up of RIGHT-2 participants to 1 year post randomisation, functional outcome did not differ between GTN and sham treatment in either the target population of stroke or TIA, or in all patients (including mimics). However, GTN was associated with reduced dependency in participants with a neurovascular mimicking condition and a non-significant increase in dependency in those with ICH. There were no differences between the treatment groups in any of the secondary outcomes.

Follow-up beyond 3 months is not typical in acute stroke trials^16–19^ but is increasingly recommended, especially in patients with severe and/or haemorrhagic stroke, where longer periods of follow-up to 6, 12 or even 18 months may be needed to see significant improvements in functional outcome and differences between treatment groups. The results presented here at 12 months after randomisation are qualitatively similar to those reported for follow-up at 3 months^11^; in particular, there were no overall differences in the functional or secondary outcomes between the GTN and sham treatment groups. However, GTN appeared to be superior to sham in mimics (mainly comprising seizures, migraine and functional events).^11^ These findings follow those shown in the prespecified subgroup analysis of patients with mimic.^20^ It is unclear why GTN should benefit mimics; this could reflect a chance finding but will need another trial to assess whether real. In contrast, GTN was associated with a non-significant worse outcome in ICH at both 3 and 12 months.^21^ The mechanism for why GTN might worsen ICH is unclear but could reflect that GTN, as a NO donor, impedes the first (vasoconstriction) and second (platelet aggregation) phases of haemostasis. A tendency for GTN to be inferior to sham in the target population of stroke or TIA at 3 months was not apparent at 12 months.^11^ Although these findings are difficult to explain and may simply reflect chance or imbalances at baseline, there are other possible explanations as explored elsewhere.^20 21^ Overall, while between-group findings were qualitatively similar at 1 year, the distribution of outcomes in both treatment groups did differ modestly as compared with 3 months, with both increased proportions of patients with low dependency levels (reflecting intervening further recovery of alive patients) and increased mortality (reflecting intervening further incident fatal events).

The strength of this secondary analysis is the near-complete follow-up at 1 year (vital status in 98% and functional outcome in 95% of the participants) in this high-fidelity trial. The high rate of long-term follow-up is reassuring in a trial where participants were recruited in ambulances in a time-limited environment in the prehospital period. Indeed, follow-up of non-stroke mimics to 12 months is novel. The main weakness is that we did not collect information on factors such as secondary prevention and rehabilitation in the community that will impact on outcome at 1 year.

In summary, GTN did not benefit patients with ultra-acute presumed stroke at 1 year when administered in the prehospital environment. Other prehospital trials of GTN (MR ASAP, ISRCTN99503308) and BP lowering (INTERACT-4, NCT03790800) will further examine the question of ultra-acute BP lowering after stroke.^22^

## Data Availability

Data are available upon reasonable request. Individual participant data will be shared with the Virtual International Stroke Trials Archive collaboration. From 1 January 2021, the chief investigator (with approval from the trial steering committee as necessary) will consider other requests to share individual participant data via email at right-2@nottingham.ac.uk. We will require a protocol detailing hypothesis, aims, analyses, and intended tables and figures. Where possible, we will perform the analyses; alternatively, deidentified data and a data dictionary will be supplied for the necessary variables for remote analysis. Any sharing will be subject to a signed data access agreement. Ultimately, the data will be published.
